# Exposure to Environmental Tobacco Smoke and Cognitive Abilities Among U.S. Children and Adolescents

**DOI:** 10.1289/ehp.113-a296a

**Published:** 2005-05

**Authors:** Phillip Petersen

**Affiliations:** Queensland Medical Laboratory and Queensland University of Technology, Rochedale, South Queensland, Australia, E-mail: philpetersen@optusnet.com.au

In the article “Exposure to Environmental Tobacco Smoke and Cognitive Abilities Among U.S. Children and Adolescents,” Yolton et al. (2005) stated that the data “indicate an inverse association between ETS exposure and cognitive deficits among children ….” They do not. They indicate an inverse association between ETS exposure and scores, but a direct association between ETS exposure and cognitive deficits.
